# Case Report: Synchronous pancreatic sarcomatoid carcinoma and gastric cancer: a case study with literature review

**DOI:** 10.3389/fimmu.2025.1584504

**Published:** 2025-05-27

**Authors:** Genlin Lu, Jinming Tu, Renya Jiang

**Affiliations:** ^1^ Department of General Surgery (Key Disciplines of Medicine in Quzhou City), Longyou County People’s Hospital, Longyou People’s Hospital Affiliated with Sir Run Run Shaw Hospital, Zhejiang University School of Medicine, Quzhou, China; ^2^ Department of Gastroenterology, Longyou County People’s Hospital, Quzhou, China; ^3^ Department of Hepatobiliary Surgery, Quzhou City People’s Hospital, Quzhou, China

**Keywords:** pancreatic sarcomatoid carcinoma, gastric cancer, surgical treatment, enteral nutrition, parenteral nutrition

## Abstract

**Objective:**

To illuminate the pathological characteristics and treatment of synchronous pancreatic sarcomatoid carcinoma(SCP) and gastric cancer using a case report and comprehensive literature review.

**Methods:**

Clinical presentation, pathological findings and immunohistochemical results of a patient with SCP and gastric cancer were reviewed. A surgical treatment strategy with best supportive care, including nutrition support, was adopted. A comprehensive literature review was conducted.

**Results:**

A 69-year-old female patient presented to the general surgery clinic with ten days of abdominal pain. A Type IIc lesion measuring 30 mm in diameter was found in the gastric angle. Additionally,a thick-walled, solid mass with dimensions of 33 mm by 30 mm was detected in the pancreatic tail. HE staining revealed a moderately to poorly differentiated adenocarcinoma and partial mucinous adenocarcinoma in gastric angle and a sarcomatoid carcinoma with low-grade adenocarcinoma components in pancreatic tail. Immunohistochemical assay of the mass in pancreatic tail showed partial positivity for CK(pan), CK7, CD56, CD138, CEA, EMA, and Ki-67 (60%). The patient’s serum levels of CA199, CEA, CA125, and CA242 were sharply decreased postoperatively but remained above normal limits. However, these markers were predominantly increased in the presence of multiple liver metastases.Malnutrition and progressive disease occurred despite the adoption of EN plus PN and best supportive care, and the patient died on postoperative day 107.

**Conclusions:**

Synchronous SCP and gastric cancer are first reported in this study. Surgical treatment remains effective despite an even poorer prognosis.

## 1 Introduction

In 2022, 968,350 patients were initially diagnosed with stomach cancer, and 659,853 died from it. Meanwhile, pancreatic cancer had 510,566 new cases and caused 467,005 deaths (1). The 5-year overall survival rate for pancreatic cancer was approximately 2-9%, due to limited treatment options (1). A total of 15 case reports on synchronous stomach cancer and pancreatic cancer have been discribed until now (2, 3) (**Table 1**). No reports on stomach cancer and pancreatic sarcomatoid carcinoma (SCP)have been found. Here is the case report and literature review.

**Table 1:** Summary of the literature on synchronous gastric and pancreatic cancer.

First author/year	Gender	Age	Treatment	Histology		Tumor location	
				Stomach	Pancreas	Stomach	Pancreas
Ebihara M 2024 (2)	male	59	Laparoscopic total gastrectomy and distal pancreatectomy+S-1	poorly differentiated adenocarcinoma	invasive ductal carcinoma	the anterior wall of the upper gastric body of the greater curvature	pancreatic tail
Eriguchi 2000 (4)	male	76	subtotal gastrectomy and distal pancreatectomy with Lymph nodes dissection	moderately differentiated tubular adenocarcinoma	Well to moderately differentiated tubular adenocarcinoma	NR	NR
Fang T2021 (5)	male	69	Distal gastrectomy and distal pancreatectomy, SOX	moderate to poorly differentiated adenocarcinoma	acinar cell carcinoma	antrum	pancreatic tail
Ghothim 2015 (6)	male male	73 74	Surgically treated,gemcitabine Surgically treated; radiochemotherapy	Adenocarcinoma Adenocarcinoma(diffuse type)	Ductal pancreatic cancer Papillary mucinous carcinoma	antrum antrum	Pancreatic head Pancreatic head
Inoue 2021 (7)	male	78	Total gastrectomy,distal pancreatectomy, splenectomy	signet ring cell carcinoma	PanIN-3	lesser curvature	pancreatic body and tail
Kourie 2012 (8)	Male male	56 62	Folfirinox Folfirinox	Poorly differentiated adenocarcinoma adenocarcinoma	ductal adenocarcinoma Tubular carcinoma	anterior part of the antrum greater curvature	pancreatic head pancreatic tail
Kubota2009 (9)	Male	67	S-1,paclitaxel and lentinan	Moderately differenciated adenocarcinoma	NR	NR	NR
Ma YH2021 (10)	female	81	ESD+Laparoscopic distal pancreatectomy, splenectomy	early gastric cancer and gastric DLBCL	Invasive ductal carcinoma	greater curvature of antrum	pancreatic tail
Muroni 2010 (11)	male	73	pancreaticoduodenectomy	a moderately differentiated adenocarcinoma	poorly differentiated ductal adenocarcinoma	antrum	uncinate portion
Ohtsubo 2013 (12)	male	77	S-1	adenocarcinoma	adenocarcinoma	In the middle of stomach	pancreatic tail
Read C2023 (13)	Female	57	FOLFIRINOX and Opdivo Subtotal pancreatectomy, splenectomy, gastrectomy	high-grade poorly differentiated gastric adenocarcinoma	well-differentiated adenocarcinoma	fundus, the greater curvature, and the lesser curvature	pancreatic body and tail
Santos-Ferná ndez 2015 (14)	Female	64	NR	Well differentiated adenocarcinoma	adenocarcinoma	Prepiloric antral ulcer	Pancreatic tail
Shupp 2022 (15)	female	76	an extended Whipple procedure chemoradiation therapy with 5-fluorouracil	signet ring cell gastric adenocarcinoma	adenocarcinoma	antrum	pancreatic head
Yonenaga 2016 (16)	male	63	Chemotherapy	Poorly differentiated adenocarcinoma	ductal adenocarcinoma	antrum	pancreatic body
Yuichiro Yokoyama 2009 (3)	Male	86	total gastrectomy distal pancreatosplenectomy	Poorly differentiated adenocarcinoma, moderately differentiated tubular adenocarcinoma	well-differentiated adenocarcinoma	antrum	pancreatic body

ESD, endoscopic submucosal dissection; NR, not report; DLBCL, Diffuse Large B-Cell Lymphoma; PanIN-3, Pancreatic Intraepithelial Neoplasia Grade 3.

The article is presented in line with the CARE reporting checklist (**Supplementary Material**).

## 2 Case presentation

### 2.1 General information

A 69-year-old female patient presented to the general surgery clinic with ten days of abdominal pain (+0 day). She experienced epigastric dull pain for 10 days, characterized by paroxysmal onset, more prominent at night and during the postprandial period, and relieved after sitting for 30 minutes. There was no radiation of pain to other areas, no acid regurgitation or belching, no nausea or vomiting, no hematemesis or melena. No risk factors for gastric cancer such as alcohol consumption, smoking, intake of salted and smoked foods, or gastroesophageal reflux disease were found. He had no history of psychosocial issues, oncological conditios, or genetic diseases, nor any relevant family history.

Upon physical examination, no signs of anemia, jaundice of the skin and sclera, enlargement of cervical or supraclavicular lymph nodes, liver palms, or spider nevi were detected. The abdomen was flat with no visible gastrointestinal or peristaltic waves, varicose veins, hepatomegaly, splenomegaly, shifting dullness, tenderness, rebound tenderness, or muscular tension.

**Figure 1:**
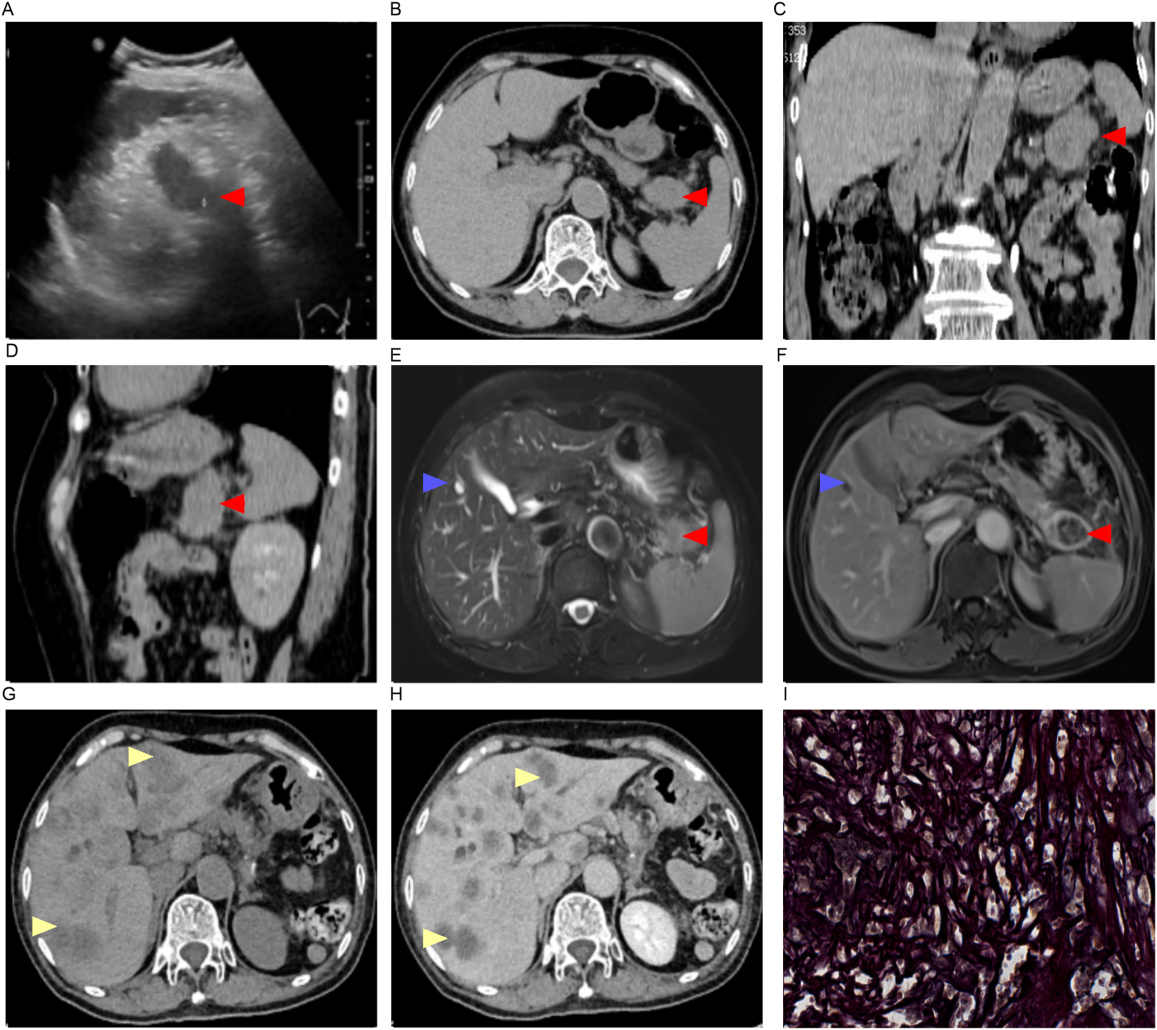
Imaging findings and pathological manifestations. **(A)** Ultrasound imaging (+0 day);**(B–D)**, CT imaging [**(C)**, coronal; **(D)**, sagittal +0 day]; **(E, F)**, MRI imaging (+1 day); **(G, H)**, CT imaging (+56 day); **(I)** Reticular fiber staining of pancreatic tail tumor (–);Red arrow, pancreatic mass; Blue arrow, hepatic cyst; Yellow arrow, liver metastasis.

**Figure 2:**
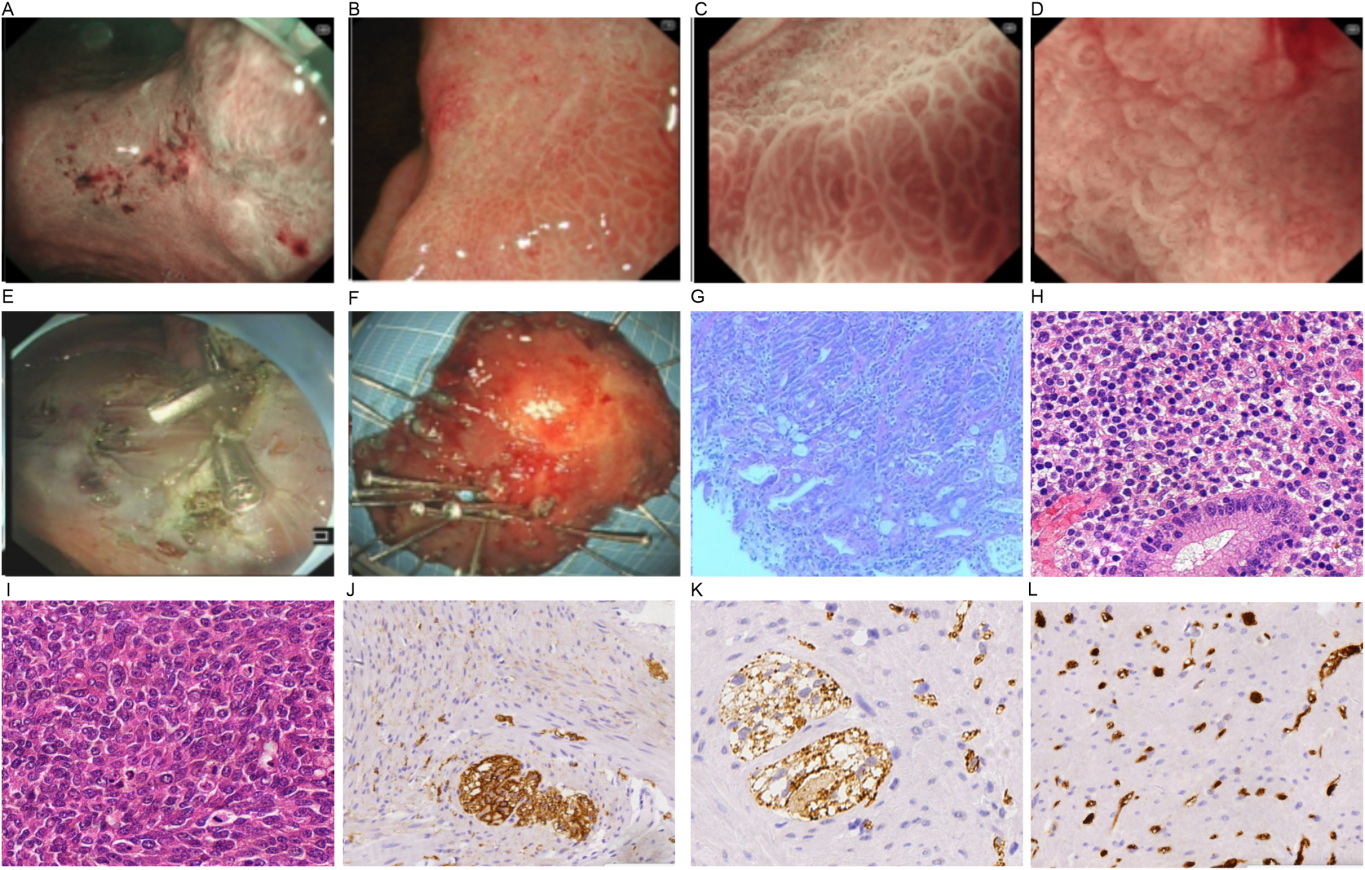
Endoscopic imaging **(A–F)** and pathological findings **(G, H)**. **(A)** lesion in the gastric antrum; **(B)** DL (+); **(C)** IMVP(+); **(D)** IMSP(+); **(E)** endoscopic imaging after ESD; **(F)** specimen from ESD; **(G–l)** pathological manifestations [**(G)** gastric biopsy under endoscopy, **(H)** specimen from ESD, **(I)** tumor in pancreatic tail]; **(J)** CD68(+); **(K)** CK (pan) (+); **(I)** Ki67 (partial +).

Sonographically, a hypoechoic mass in the splenorenal space measured 33 mm by 30 mm. The lesion was poorly defined, with neither obvious motility nor significant blood flow signals (+0 day, **Figure 1A**). A CT scan demonstrated a low-density lesion in the pancreatic tail (+0 day, **Figures 1B–D**). Under gastroscopy, a Type IIc lesion measuring 30 mm in diameter was found on the posterior wall at the gastric angle.It showed an irregular microvascular pattern (IMVP), an irregular microsurface pattern (IMSP), and a distinct boundary line (DL)(+0 day, **Figures 2A–D**). Biopsies were taken with cold forceps for histology (+0 day).

The patient was admitted to the general surgical department with synchronous lesions: early gastric cancer at the gastric angle (T_1_N_0_M_0,_ Stage I) and a pancreatic mass (+0 day).

**Figure 3:**
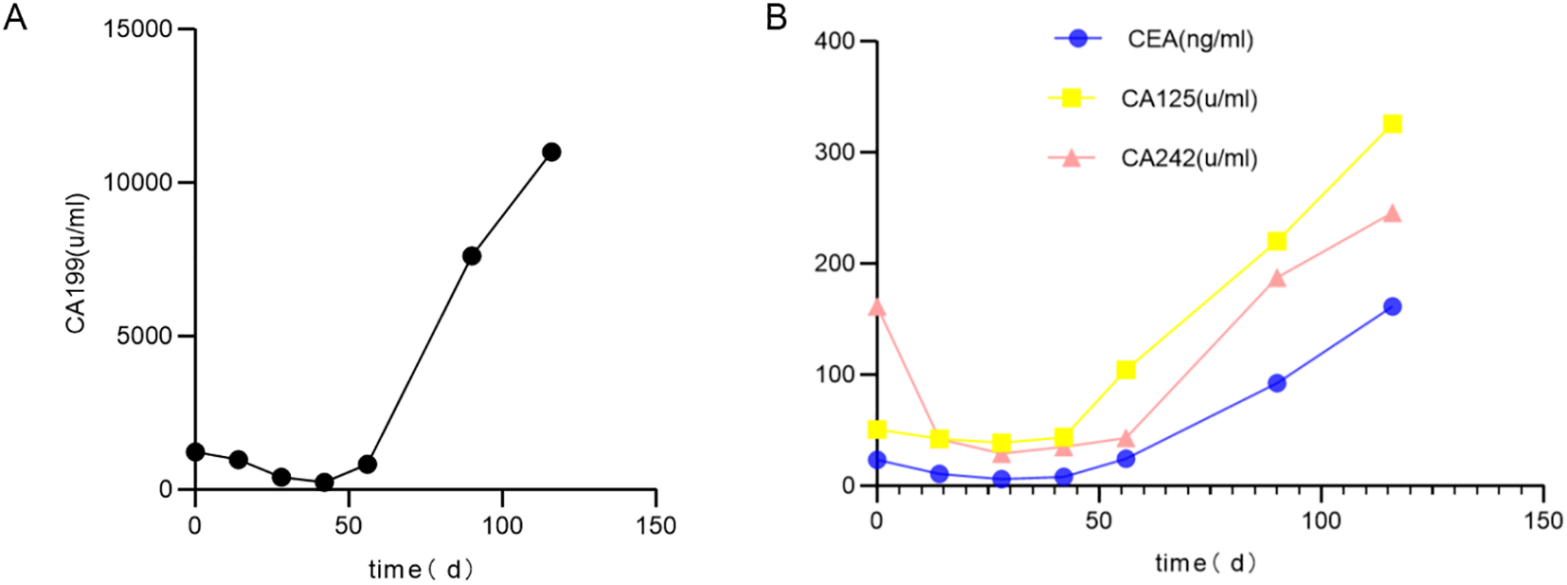
Change of tumor biomarkers. **(A)** CA199; **(B)** CEA, CA125, CA242.

On Day 0, the serum levels of CA199, CEA, CA125, and CA242 were elevated (**Figures 3A, B**). The levels of CA125, AFP, CA724, CA50, and CA153 were found to be within normal ranges. The contrast -enhanced MRI identified a 21 mm intrahepatic cyst and a thick-walled, solid mass in the pancreatic tail. The mass measures 33 mm by 30 mm with mild to moderate heterogeneous enhancement. As such, a solid neoplasm in the pancreatic tail and a hepatic cyst were initially considered. (+1 day, **Figures 1E, F**). No enlarged lymph nodes around the stomach, pancreas, spleen or in postperiternium were delineated in ultrasonography, CT, and MRI (**Figures 1A–F**).

### 2.2 Treatment

**Figure 4:**
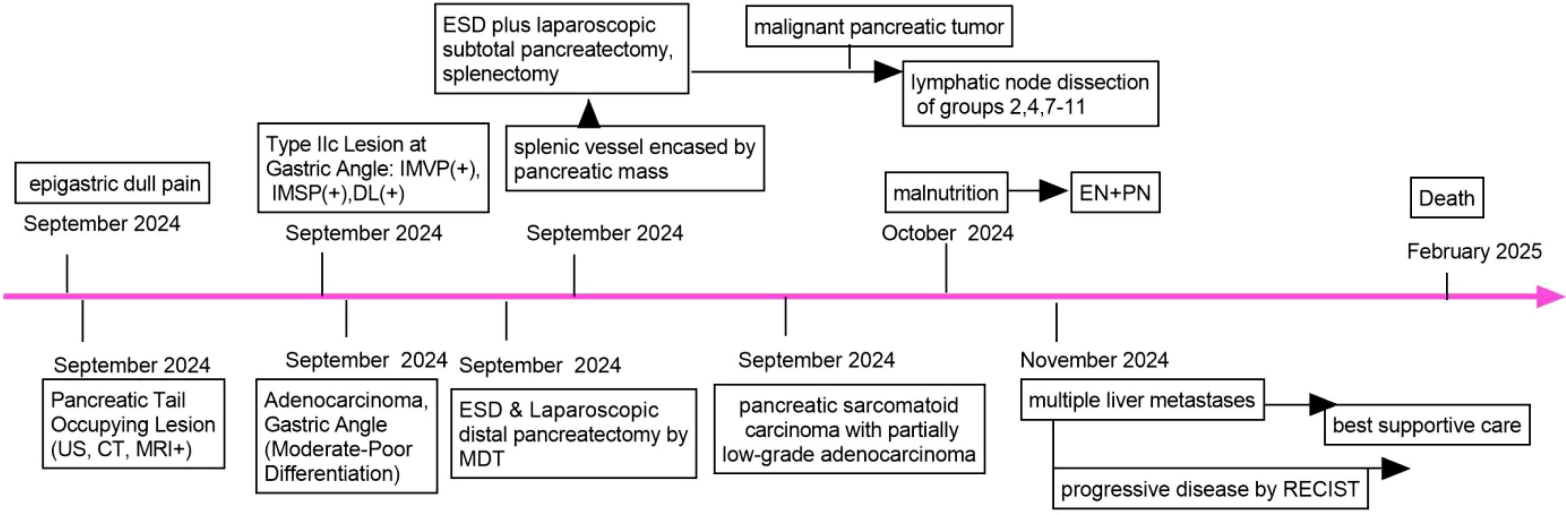
Timeline of the case report. US, ultrasound; CT, computer tomography; MRI, magnetic resonance imaging; EN, enteral nutrition; PN, parenteral nutrition; ESD, endoscopic submucosal dissection; IMVP, irregular microvascular pattern; IMSP,irregular microsurface pattern; DL, distinct boundary line; MDT, Multidisciplinary Discussion; RECIST, response evaluation criteria in solid tumors.

According to the eighth edition of TNM classification system for gastric carcinoma (17), the patient was preoperatively staged as T_1_N_0_M_0_ (stomach cancer) and a solid neoplasm in pancreatic tail. The treatment strategy was confirmed to be gastric endoscopic submucosal dissection (ESD) combined with laparoscopic resection of the pancreatic tail and body, as determined by a multidisciplinary discussion. (+7 days, **Figure 4**).

The patient underwent general anesthesia (+9 days). Upon ME-NBI observation, a 3.0 cm × 3.0 cm lesion within the resected area at the gastric angle was noted as IMSP+, IMVP+, and DL+ (**Figures 2B–D**). As depicted in **Figures 2E, F**, the gastric angle submucosa was resected during ESD, measuring 5.0 cm by 4.0 cm. During laparoscopic exploration, the splenic vessel was encased within the pancreatic mass, making it quite difficult to isolate. No intra-abdominal dissemination was found, and ascites cytology was negative. After obtaining informed consent from the patient’s family, laparoscopic subtotal pancreatectomy and splenectomy were performed (**Figure 4**).

Histologic examination of intraoperative frozen section indicated pancreatic malignancy (**Figure 2I**). The transection margin was free of tumor. Lymph nodes in groups 2, 4, 7–11 were laparoscopically cleared (**Figure 4**). No metastasis was reported in lymph nodes.

Enteral nutrition (EN) plus parenteral nutrition (PN) was administered when malnutrition occurred (+24 days, **Figure 4**). It is detrimental that the patient has rejected adjuvant therapy, including chemotherapy. Given that the disease had metastasized to the liver, making curative treatment unfeasible, best supportive care was provided (+56 days, **Figure 4**).

### 2.3 Outcome

Contrast-enhanced CT revealed multiple hepatic nodular hypodense lesions with a bull’s-eye appearance, which indicated a progressive disease stage according to the RECIST criteria (+56 days, **Figures 1G, H**). The patient passed away on February 1, 2025 (+116 days, **Figure 4**).

## 3 Result

### 3.1 Changes of tumor biomarkers

As shown in **Figures 3A, B**, the serum levels of CA199, CEA, CA125, and CA242 were elevated above normal range before the operation. These markers decreased sharply after the operation but remained abnormal. Subsequently, their levels increased predominantly in association with liver metastasis.

### 3.2 Pathological findings

Analysis of a biopsy sample from the gastric angle demonstrated chronic superficial antral gastritis, moderate intestinal metaplasia, partial disruption of the glandular structure, and the presence of atypical cells in focal stromal areas (**Figure 2G**).

As displayed in **Figure 2H**, the pathological result of the ESD specimen showed a moderately to poorly differentiated adenocarcinoma. The findings included partial mucinous adenocarcinoma, intestinal metaplasia, and an invasion depth of the submucosa, 0.22 cm away from the muscularis mucosae. The resection margins were negative, and there was no neural or vascular invasion (+16 days).

As illustrated in **Figure 2I**, the mass in the pancreatic tail was composed of spindle-shaped cells without a glandular component and was diagnosed as a sarcomatoid carcinoma with low-grade adenocarcinoma components. It exhibited neural invasion but neither lymphatic invasion nor vessel invasion.

The postoperative TNM classification is T_1a_N_0_M_0,_Stage I (stomach cancer) and T_2_N_0_M_0,_Stage IB(SCP) (17, 18).

### 3.3 Immunohistochemical results

The gastric lesion stained positively for CK(pan), CD68, and was partially positive for Ki-67 (**Figures 2J–L**).

**Figure 5:**
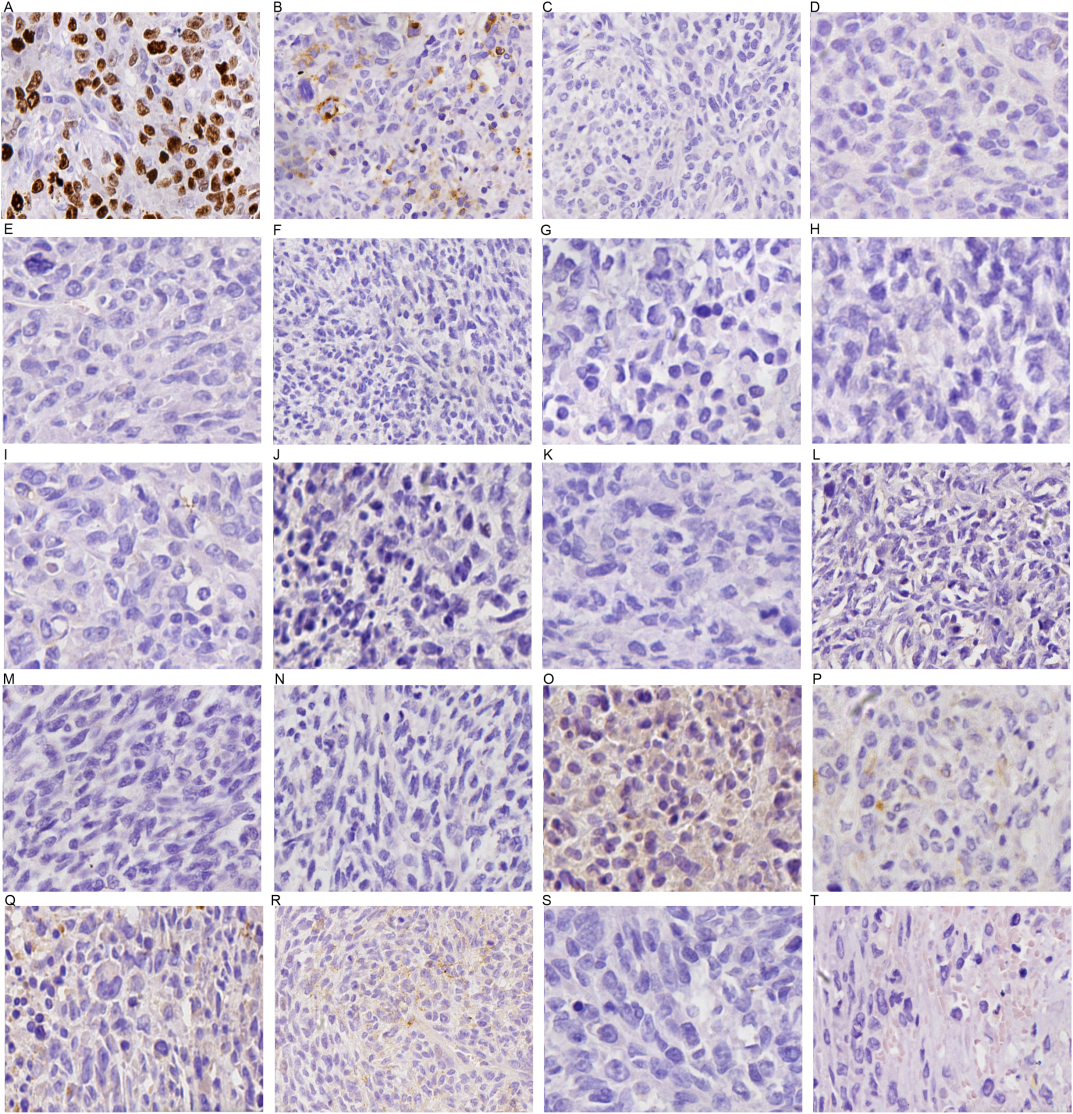
IHC result of the mass in pancreatic tail. **(A)** Ki67 (60%+); **(B)** CD56 (partial +); **(C)** P40 (–): **(D)** TTF-1 (–); **(E)** Syn (–); **(F)** CgA (–); **(G)** CD117 (–); **(H)** S-100 (–); **(I)** DOG-1 (–); **(J)** CD34 (–); **(K)** HMB45 (–); **(L)** Myogenin (–); **(M)** SMA (–); **(N)** Desmin (–); **(O)** CEA(partial +); **(P)** EMA(partial+); **(Q)** CK7(partial +); **(R)** CD138 (partial +), **(S)** P63 (–) **(T)** CK (pan) (partial+).

The mass in the pancreatic tail was partially positive for CK(pan), CK7, CD56, CD138, CEA, EMA, and Ki-67 (60%), but negative for P40, P63, CgA, Syn, CD117, DOG1, S-100, HMB45, CD34, SMA, Desmin, Myogenin, TTF-1, and reticular fiber staining (+16 days, **Figures 5A–T**, **Figure 1I**).

Taken together, the IHC test supported a diagnosis of synchronous gastric adenocarcinoma and SCP.

## 4 Discussion

On the basis of the GLOBOCAN data, gastric cancer ranks 5th and pancreatic cancer ranks 12th in terms of incidence (1). Regarding mortality rate, gastric cancer is the 5th leading cause of cancer- related deaths, while pancreatic cancer ranks 6th (1). As summarized in **Table 1**, the average age of patients with synchronous gastric and pancreatic cancer is 70.06 ± 8.81 years. The female proportion is 23.5%. Nevertheless, it is widely accepted that there are no reports in the literature on synchronous gastric cancer and SCP to date. This study highlights a rare case involving a 69-year-old female patient with this unusual combination.

SCP constitutes a variant of undifferentiated pancreatic carcinoma and is associated with an extremely poor prognosis (19). Radical resection and adjuvant therapy, such as gemcitabine plus capecitabine, are mainly effective for this rare but deadly disease (20). In the context of sarcomatoid features, both mesenchymal and epithelial markers are typically co-expressed (19). Immunohisto- chemical markers are helpful in the differential diagnosis of pancreatic tumors. Gastrointestinal stromal tumor (GIST) was ruled out due to the negativity of CD117, DOG1, CD34, and SMA. The negativity of CgA and Syn enabled us to exclude pancreatic neuroendocrine tumors (pNETs). Negative staining for S-100 and HMB45 supported the exclusion of pancreatic malignant melanoma. Negative results for P40, P63, and TTF-1 aid in eliminating pancreatic metastasis of pulmonary squamous cell carcinoma and pulmonary adenocarcinoma. Negativity for Desmin and Myogenin staining typically precludes malignant tumors of skeletal muscle origin. A 60% positivity rate for Ki-67, partial positivity for CK(pan), CK7, CEA, EMA, CD56, and CD138, along with the pathological features, support the diagnosis of SCP with adenocarcinoma components.

Drawing on the histological findings and IHC results, the patient was diagnosed with synchronous gastric adenocarcinoma (T_1a_N_0_M_0_) and SCP (T_2_N_0_M_0_). At the same time, the following tumors should be differentiated:

1. Pancreatic invasion by gastric cancer. In this setting, the lesion is generally located in the greater curvature or posterior wall of the stomach. No clear space between these structures and enlarged lymph nodes surrounding the stomach was usually found on CT or MRI scans. The expressions of HER2, MLH1, MSH2, PMS2, MSH6, Ki67, and PD-L1 in the IHC test help us to identify this disease.
2. Pancreatic cancer metastasizing to stomach. In this scenario, encasements between the two organs were always noted on CT or MRI scans. No lesion was normally noticed in the gastric mucosa under gastroscopy. The positivity of CEA, CA19-9, MUC-1, CK-19, and Ki67 makes this diagnosis appropriate.

There is no consensus on treatment options for synchronous gastric and pancreatic cancer. As outlined in **Table 1**, both the surgically-treated strategy and Fluorouracil-based chemotherapy have been shown to be effective in treating simultaneous gastric and pancreatic cancer (2, 4–13, 15, 16). Ebihara M, et al. suggested that laparoscopic radical total gastrectomy and pancreatosplenectomy are preferred for the treatment of concurrent cancer of the stomach and pancreas (2). It was generally acknowleged that surgical resection should be conducted for pancreatic tail masses larger than 30 mm. In this study, to avoid the risk of SCP metastasizing to the stomach, endoscopic ultrasound-guided biopsy was not performed (16). Since Type IIc is a kind of early gastric cancer, ESD is considered appropriate. Considering the malignancy in the pancreatic tail, laparoscopic subtotal pancreatectomy, splenectomy, and lymph node resection were ultimately conducted.

The rate of postoperative pancreatic fistula (POPF) is about 23%, which is a severe complication during pancreatic surgery and a significant challenge for surgeons (21). A soft pancreatic texture and a main pancreatic duct (MPD) diameter of no more than 3 mm are considered objective pancreatic- specific factors associated with an increased risk of POPF (21). Intraoperative ultrasonography provides precise information about pancreatic texture and MPD size for surgeons, which offers a real-time, comprehensive evaluation of pancreatic anatomy when laparoscopic pancreatectomy is performed. It is intriguing that an innovative intraoperative Wirsung duct high-quality ultrasonography(IWU) has been utilized to assess the structural characteristics of the main pancreatic duct (22). IWU can also be used as a mitigation strategy by evaluating the MPD during laparoscopic distal pancreatectomy. Only laparoscopic exploration was performed during the operation in this study. No other pancreatic lesions were detected, which was postoperatively verified by contrast-enhanced CT, and no POPF developed.

Compared to pancreatic ductal adenocarcinoma, SCP has a worse prognosis, with a median overall survival of 6 months (23). Gemcitabine plus capecitabine chemotherapy was postoperatively administered as adjuvant therapy for SCP, and the patient was reported to be alive 3 months after the operation (20). Since the patient refused adjuvant therapy, including chemotherapy, EN plus PN was administered when malnutrition manifested. Best supportive care was adopted as progressive disease occurred. The patient died on the 107th postoperative day.

## 5 Conclusion

A rare case, with a comprehensive literature review, demonstrates compelling evidence that positivity for CK(pan), CK7, CD56, CD138, CEA, EMA, and Ki-67 (60%) in spindle-shaped cells without a glandular component contributes significantly to diagnosing SCP with low-grade adenocarcinoma components. Furthermore, this study reports the first case of synchronous SCP and gastric cancer. Minimally invasive surgical treatment, including ESD and laparoscopic radical pancreatosplenectomy, remains effective despite an even poorer prognosis.

## Data Availability

The original contributions presented in the study are included in the article/supplementary material. Further inquiries can be directed to the corresponding author.
